# Research advances in the influence of lipid metabolism on cognitive impairment

**DOI:** 10.1002/ibra.12018

**Published:** 2022-02-18

**Authors:** Min Zhang, Yu‐Hang Zhu, Zhao‐Qiong Zhu

**Affiliations:** ^1^ Department of Anesthesiology Affiliated Hospital of Zunyi Medical University Zunyi Guizhou China; ^2^ Suining Central Hospital Suining Sichuan China

**Keywords:** cognitive dysfunction, hyperlipidemia, lipid metabolism

## Abstract

Cognitive impairment (CI) is a mental disorder related to cognition and understanding, which is mainly categorized into mild CI and senile dementia. This disease is associated with multiple factors, such as chronic brain injury, aging, chronic systemic disease, mental state, and psychological factors. However, the pathological mechanism of CI remains unclear; it is usually associated with such underlying diseases as diabetes and hyperlipidemia. It has been demonstrated that abundant lipid metabolism indexes in the human body are closely related to CI, including total cholesterol, high‐density lipoprotein cholesterol, low‐density lipoprotein cholesterol, triglycerides, apolipoprotein, and so forth. As a crucial risk factor for CI, hyperlipidemia is of great significance in the occurrence and development of CI. However, the specific correlation between dyslipidemia and CI is still not fully elucidated. Besides, the efficacy of lipid‐lowering drugs in the prophylaxis and treatment of CI has not been clarified. In this study, relevant advances in the influence of lipid metabolism disorders in CI will be reviewed, in an attempt to explore the effect of mediating blood lipid levels on the prophylaxis and treatment of CI, thus providing a reference for its clinical management.

## Graphical abstract

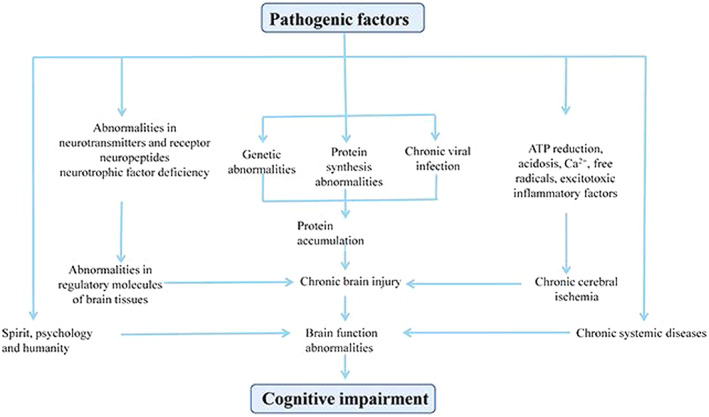

## 1 INTRODUCTION

Cognitive impairment (CI) is the impairment of at least one cognitive function with respect to memory, language, visual space, calculation, execution, and comprehension judgment, which significantly affects daily life and social abilities.^1^, ^2^ CI is mainly categorized into mild cognitive impairment (MCI) and senior dementia.^3^, ^4^ In general, this disease is mainly caused by chronic brain injury, aging, chronic systemic disease, mental state, and psychological factors, despite the fact that the exact pathogenesis remains unclear. It has been demonstrated in previous evidence that aging is the independent high‐risk factor for CI.^5^, ^6^ Besides, as confirmed in abundant international population studies and surveys, the prevalence of CI in the population 60 years of age or older ranges from 15% to 20%, and the annual rate of MCI developing into dementia is up to 8%–15%, which astonishingly alarms medical practitioners worldwide that CI should be concerned and treated in an individualized manner.^7^ With an increasing trend of population aging at home and abroad, the rapidly increased incidence of CI not only affects the quality of life of the elderly but also poses a huge burden on the family, medical care, and social economy.

With the aging population and the improved quality of life, the risk of obesity and other metabolic syndromes (such as hyperlipidemia and diabetes)^8^ and mental illnesses (such as depression^9^ and anxiety^10^) has been increasing. It has been demonstrated in recent studies that there is a direct correlation between hyperlipidemia and CI. The long‐term lipid metabolism disorder is the main cause of hyperlipidemia, which is considered a risk factor for inducing and aggravating CI.^11^, ^12^ Metabolic indexes, such as total cholesterol (TC), triglycerides (TGs), apolipoprotein (Apo), and lipoprotein a (Lp(a)), play a significant role in the occurrence and development of CI. In this study, the research advances on the influence of lipid metabolism disorders on CI will be reviewed, which can be useful for the clinical prophylaxis and treatment of CI by regulating blood lipid levels.

## 2 OVERVIEW OF LIPID METABOLISM DISORDERS

**Figure 1:**
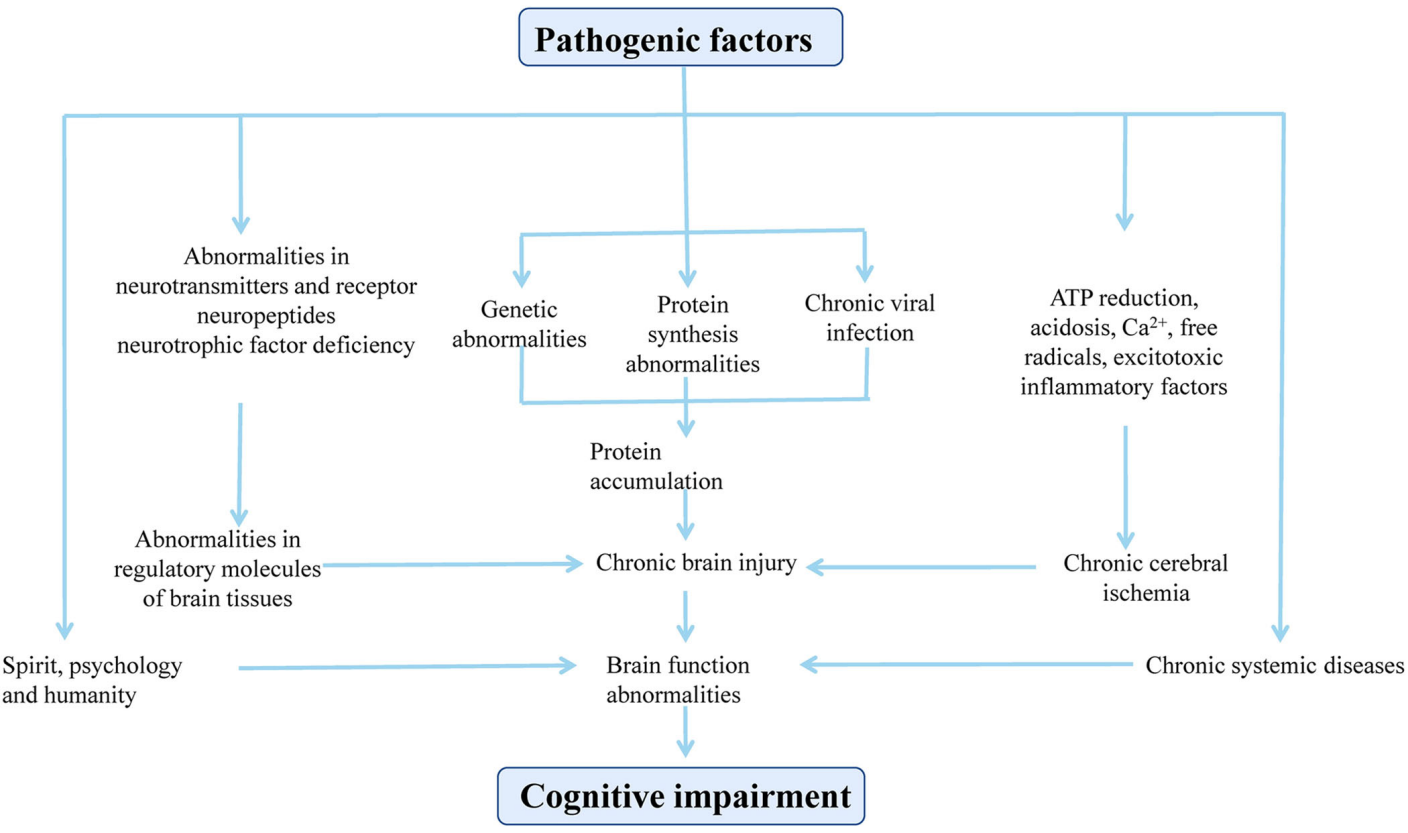
Etiology and pathogenesis of cognitive impairment. Any factor that directly or indirectly leads to chronic damage of cerebral cortex structure and function can cause cognitive impairment through different mechanisms. It mainly includes the following aspects: chronic brain injury caused by various pathogenic factors, chronic systemic diseases, mental and psychological abnormalities, and the influence of human factors [Color figure can be viewed at wileyonlinelibrary.com]

Lipid metabolism disorders refer to the quantitative and qualitative abnormalities of lipids and their metabolites in the blood and organs caused by congenital or acquired factors. It can be categorized into primary and secondary lipid metabolism disorders. The former subtype is closely related to congenital and genetic factors, such as the single‐gene or multigene mutation of receptors, enzymes, or carriers involved in lipoprotein transport and metabolism. Besides, it can also be induced by environmental factors (such as diet, nutrition, and drugs) or unknown mechanisms. However, the latter subtype mostly occurs following metabolic disorders (such as diabetes, hypertension, obesity, liver, and kidney diseases) or is influenced by other factors, like age, gender, diet, physical activity, and mental stress.^13^, ^14^ Hyperlipidemia is an abnormal systemic lipid metabolism caused by increased plasma TC, TG, and low‐density lipoprotein cholesterol (LDL‐C), as well as decreased high‐density lipoprotein cholesterol (HDL‐C). Usually, there are no typical symptoms and signs in patients with hyperlipidemia, who are often diagnosed during physical examinations or clinical visits for relevant diseases. Therefore, hyperlipidemia is easily overlooked in daily life.^15^ It is reported that hyperlipidemia can reduce the activity of free radical scavengers, which would lead to a massive accumulation of lipid peroxides, thus accelerating the development of atherosclerosis. Meanwhile, the reduction of cerebral blood flow causes cerebral ischemia and hypoxia, and ultimately leads to brain damage and CI.^16^, ^17^ Moreover, it has been confirmed in recent studies that abnormally expressed LDL‐C, apolipoprotein A (Apo A), and apolipoprotein B (Apo B) have a close relationship with CI.^18^, ^19^, ^20^ Lipid metabolism disorders are common chronic diseases in the elderly, which eventually cause coronary heart disease and cerebrovascular disease. Besides, they are independent risk factors for MCI in the elderly. Hence, considerable attention should be paid to lipid metabolism disorders^21^ (Figure 1).

## 3 RESEARCH ON THE INFLUENCE OF CHOLESTEROL IN CI

Cholesterol is present extensively in the brain, nerves, and other tissues. High levels of cholesterol in the blood are likely to induce lipid plaques that will affect the blood supply of target organs and cause tissue ischemia and hypoxia. As a result, tissue structure and function are damaged.

### 3.1 TC and CI

Increased TC is an important factor for the occurrence and development of CI. Based on a literature review, the underlying mechanisms of increased TC to cause CI were reported as follows. First, high levels of TC affect cerebral blood supply and induce lipid plaques in the carotid artery, which increase the risk of plaque rupture. Meanwhile, the increased deposition of amyloid β‐protein (Aβ) caused by high levels of TC further influences cerebral blood supply, which causes cerebral ischemia and hypoxia, thus affecting cognitive function.^22^ Second, high levels of TC damage the blood–brain barrier (BBB). Under normal circumstances, cholesterol cannot pass through the BBB. However, high levels of TC lead to an increase in its metabolites, including 24S‐hydroxycholesterol and 27S‐hydroxycholesterol, both of which result in damage to the central nervous system and CI through damage to the BBB, inducing the penetration of serum cholesterol and inflammatory factors.^23^ Third, high levels of TC induce oxidative stress, which damages neuron cell membranes.^24^ It is speculated that the increase in TC levels triggers CI through multiple mechanisms. It is recommended to actively control TC levels, restore the cerebral blood supply, protect the BBB, and reduce oxidative stress, which contributes to alleviating hypercholesterolemia‐induced CI.

### 3.2 HDL‐C and CI

HDL‐C can transport cholesterol from extrahepatic tissues to the liver for metabolism, which converts it into bile acids or directly excretes it from the intestines through the bile. HDL‐C can also absorb cholesterol from the cell membrane to form cholesterol ester by catalysis via lecithin cholesterol acyltransferase, and then transfer it to very‐low‐density lipoprotein and low‐density lipoprotein. The plasma content of HDL‐C is negatively correlated with the risks of atherosclerosis, and cardiovascular and cerebrovascular diseases.^25^ It has been confirmed in a previous study that the plasma HDL‐C levels of CI patients are significantly lower than those of healthy controls, with a consistent level corresponding to the disease severity, which suggests an obvious correlation between plasma HDL‐C levels and CI.^26^ In a nutshell, the following reasons contribute toward the protective effect of HDL‐C on CI. First, HDL‐C can inhibit the formation of lipid plaques in the carotid artery through the antioxidative stress response, which maintains cerebral blood supply, thereby mitigating the cognitive dysfunction caused by ischemia and hypoxia.^27^ Second, HDL‐C has a fibrinolytic effect in inhibiting the formation of atherosclerotic plaques, regulating blood rheology, and reducing the blockage of cerebral blood vessels caused by lipid accumulation, thereby reducing the risk of CI.^28^ Third, HDL‐C has an antithrombotic effect that relaxes the vascular smooth muscle by regulating nitric oxide (NO) and platelet‐activating factors. In addition, it suppresses platelet aggregation and leukocyte adhesion in the vascular endothelium, which is beneficial to the prophylaxis of carotid artery thrombosis and the protection of neural and cognitive functions.^29^ Therefore, the measurement of HDL‐C levels is of great significance in the prophylaxis and management of CI.

### 3.3 LDL‐C and CI

LDL‐C is the main lipoprotein in fasting plasma, accounting for about 2/3 of plasma lipoproteins. It is transformed from very‐low‐density lipoprotein cholesterol (VLDL‐C) and is the main vehicle for transporting cholesterol to extrahepatic tissues. Increased LDL‐C is also a risk factor for the occurrence and development of CI. It is reported that increased LDL‐C may be the initiating factor for chronic inflammation of the carotid artery and a vital pathogenic factor for atherosclerosis.^30^ The elevated level of LDL‐C is positively correlated with the risk of cerebral hypoperfusion and brain microvascular injury. In addition, the elevated level of LDL‐C also causes the accumulation of lipid peroxides, which can exacerbate platelet aggregation, thus leading to increased blood viscosity, reduced cerebral blood flow, and enhanced risk of CI.^31^ According to a previous report,^32^ the elevated serum levels of TC and LDL‐C are positively related to the density of nerve plaques, which may be related to the occurrence of CI. As revealed in a study^33^ involving elderly women with coronary heart disease, high serum levels of TC and LDL‐C are related to low cognitive scores and CI. Therefore, reduction of LDL‐C levels represents a promising method for the prophylaxis of CI.

## 4 TG AND CI

TG is a lipid component involved in energy storage and transportation. Serum TG is mainly generated from intestinal absorption and liver synthesis, and it is less than 1.70 mmol/L in the healthy state. A high level of TG may induce atherosclerosis, coronary heart disease, and other ischemic cardiovascular diseases.^34^ At present, the involvement of TG in the occurrence of CI remains controversial.^35^ It is suggested that TG is not only related to the destruction of the BBB but also has a close correlation with inflammatory markers, both of which are potential factors for cognitive decline. Therefore, TG levels may be potentially related to CI.^36^ On the basis of the detection of TG in human cerebrospinal fluid by Banks et al.,^37^ radioactive labeling TG could cross the mouse BBB. An animal experiment reveals that TG could cause cognitive decline by damaging the long‐term enhanced *N*‐methyl‐d‐aspartic acid in the hippocampus.^38^ Reduction of TG levels could reverse CI and alleviate oxidative stress in the brain. High levels of TC and TG in the middle‐aged population have been confirmed to be related to cognitive decline in the elderly.^39^ Moreover, it has been demonstrated in another cross‐sectional study involving elderly patients older than 80 years of age that normal levels of TG reduced the incidence of cognitive impairment.^40^ However, it has been found that there is no correlation between TG and memory. According to the findings of a longitudinal study involving 326 women with a follow‐up period of 8 years by Henderson et al.,^41^ there is no significant correlation between TG and memory.

To sum up, the potential involvement of TG in CI remains controversial; this may differ in terms of the primary disease, disease severity, and detection methods of TG.

## 5 Apo, Lp(a), AND CI

Apo is also a common lipid metabolism index in clinical practice. It exerts significant effects on the binding and transportation of lipids, maintenance and stabilization of lipoprotein structures, recognition of lipoprotein receptors, and regulation of lipoprotein metabolism enzyme activities. Apo mainly includes Apo A, Apo B, and Apo E. Apo A is mainly responsible for regulating HDL‐C, which can transport the adjacent fat to the liver for metabolism, exerting an antiatherosclerotic effect.^42^ It is reported that Apo A can activate lecithin cholesterol acyltransferase, inhibit lipid deposition in the inner membrane, and remove accumulated cholesterol in smooth muscle cells, thereby preventing CI caused by cerebral ischemia.^43^, ^44^ Apo B can stimulate the lipidation of cholesterol in macrophages and promote the formation of foam cells, which would cause carotid arteritis and atherosclerosis. Apo B is the main Apo of LDL‐C and is positively correlated with the level of LDL‐C. It has been revealed from previous evidence that a high level of Apo B leads to a high level of LDL‐C and a high risk of CI.^45^, ^46^ Apo E is a vital protein involved in brain cholesterol transport, and it exerts effects on the lipid transport and repair of nerve tissue damage. All three subtypes of Apo E are closely related to the occurrence of CI.^47^ Apo E can affect the accumulation and clearance of Aβ, the deposition of which markedly damages the hippocampus and induces Alzheimer's disease (AD).^48^ It has been reported in a gene polymorphism study that the Apo E gene polymorphism is significantly related to susceptibility to MCI. The Apo E ε4 allele dose‐dependently increases the risk of MCI, while Apo E ε2/ε3 exert slight protective effects on MCI.^49^ Besides, it has been confirmed from accumulating evidence that Apo E ε4 is the main risk factor for AD.^50^, ^51^ In animals with hypercholesterolemia induced by a high‐fat diet, it is found that upregulated Aβ and Apo E in the temporal and frontal cortex are consistent with Aβ‐related pathological changes in AD cases.^52^ At present, it is necessary to make efforts to further explore the mechanism of the Apo gene polymorphism involved in the occurrence of CI.

Lp(a) is an independent lipoprotein that is mainly composed of LDL‐C linking to Apo B‐100 and Apo A. The level of Lp(a) is determined by genetics, rather than gender, age, diet, and other factors. Besides, it is not correlated with other lipoproteins and Apo levels, but is closely related to the occurrence of CI.^53^ Lp(a) is involved in the regulation of the fibrinolytic system, repair of cells and tissues, promotion of platelet activation and aggregation, and cerebral blood perfusion, which can increase the risk of CI.^54^

## 6 LIPID METABOLISM DISORDERS IN VASCULAR COGNITIVE IMPAIRMENT (VCI)

VCI is a syndrome involving all forms of CI from MCI to dementia, which is caused by the risk factors of cerebrovascular diseases (such as hypertension, diabetes, and hyperlipidemia) and  dominant (such as cerebral infarction and cerebral hemorrhage) or nondominant cerebrovascular diseases (such as leukopenia and chronic brain ischemia).^55^ The incidence of cerebrovascular disease and dementia in China has been on the rise in recent years. The overall prevalence of MCI in the elderly older than 65 years of age is 20.8%, among which MCI cases caused by cerebrovascular diseases and vascular risk factors account for 42.0%. Among the elderly older than 65 years of age, the prevalence of vascular dementia (VaD) is 1.50%, and VaD has become the second most common cause of dementia that is second only to AD.^56^ So far, there is a lack of consensus on the diagnostic standard of VCI. The diagnosis of VCI mainly relies on the determination of CI symptoms, vascular factors, and their causal relationship, as well as the exclusion of other CI‐related diseases.

**Figure 2:**
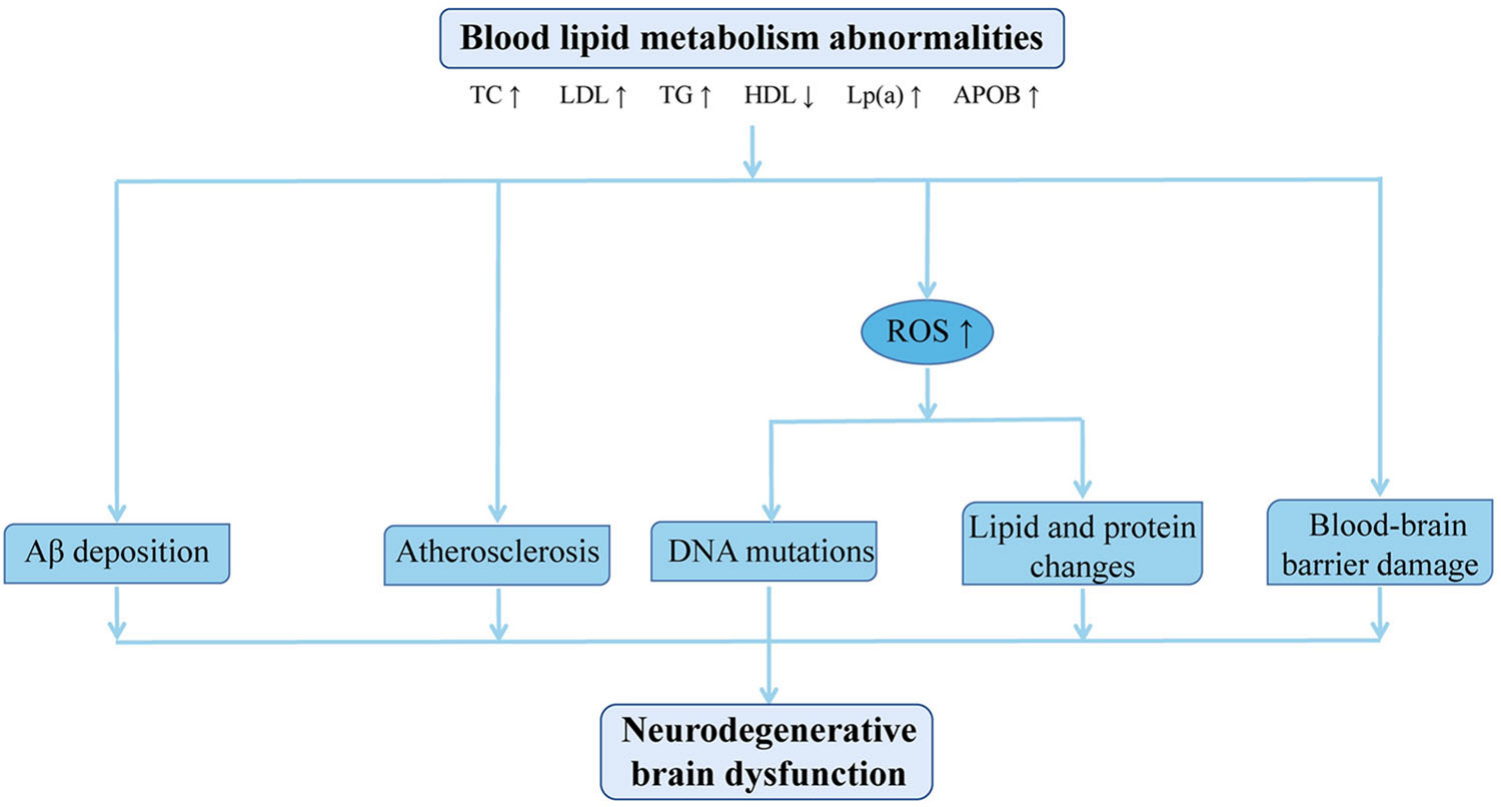
Underlying mechanism of brain dysfunction caused by abnormal lipid metabolism. Aβ, amyloid β‐protein; Apo B, apolipoprotein B; HDL, high‐density lipoprotein; LDL, low‐density lipoprotein; ROS, reactive oxygen species; TC, total cholesterol; TG, and triglyceride [Color figure can be viewed at wileyonlinelibrary.com]

A long‐term high‐fat diet can increase blood lipid levels, which would further induce cerebral atherosclerosis, blood vessel wall damage, stenosis or occlusion of the lumen, and chronic cerebral ischemic injury.^57^ Meanwhile, it thickens the cerebral artery intima, weakens cerebral vascular endothelial function, damages brain metabolism, and promotes neuronal degeneration or apoptosis. The elevated level of blood lipid caused by a high‐fat diet also increases the permeability of the BBB, upregulates microvascular endothelial cytokine VIII, induces inflammatory response, and affects cognitive function. As shown in an animal experiment,^58^ a diet rich in saturated fat and cholesterol can increase the permeability of the BBB and induce CI in mice. In addition, abnormal blood lipid metabolism downregulates fibroblast growth factor‐21 (FGF‐21) and basic FGF (bFGF), weakens their function of repairing endothelial cells, damages blood vessels and nerves, and causes learning and memory impairments.^59^ According to another study,^60^ a high‐fat diet can lead to excessive production of circulating free fatty acids, systemic inflammation, and local inflammation. CI is related to chronic and low‐level inflammatory stress, which contributes to cell‐mediated immunity that promotes an oxidative microenvironment.^61^ As revealed in a long‐term follow‐up study on elderly participants with normal cognition by Maillard et al.,^62^ elderly patients with hyperlipidemia may develop brain white matter damage with increasing age, and thus there is an increase in the risk of developing dementia. Therefore, maintaining normal blood lipid levels can contribute toward reducing the incidence of cardiovascular and cerebrovascular events, thus reducing or delaying the occurrence of AD (Figure 2).

## 7 LIPID METABOLISM DISORDERS AND INTESTINAL MICROFLORA DISORDERS

The gut flora of healthy individuals is a vast and complex ecosystem, including abundant bacteria, archaea, eukaryotes, and viruses, collectively known as gut microbes. Normal intestinal flora serves physiological functions in the host, such as digestion, absorption, nutrition, and biological antagonism. In long‐term coexistence and development, the intestinal flora and the human body form a dynamic balance that is characterized by interdependence, mutual benefit, mutual coordination, and mutual restriction, which plays an important role in the normal digestive function of the human body.^63^ In recent years, it has been suggested in a study that lipid metabolic disorders can induce dysbacteriosis; besides, it has been confirmed in a rat experiment that rats with significantly higher serum triglyceride and TC levels have an increased number of Enterobacteriaceae and a decreased number of *Bifidobacterium*, *Lactobacillus*, and *Enterococcus* in their intestines. The reason is that there are changes in the intestinal environment (such as physicochemical properties and material structure) when hyperlipidemia occurs, and these changes can affect the metabolism, growth, and reproduction of the flora, which can induce significantly decreased numbers of *Bifidobacterium*, *Lactobacillus*, and *Enterococcus*, while the number of Enterobacteriaceae remains stable and increases relatively due to less influence from the environmental changes, thus inducing dysbacteriosis.^64^ Moreover, it has been revealed in other studies that intestinal flora can affect the development and function of the nervous system. In a study of perioperative cognitive dysfunction, it has been found that intestinal microbiome dysregulation can affect cognition, mood, and behavior by regulating the immune system, vagus nerve, intestinal endocrine system, and microbiome metabolites.^65^ Therefore, lipid metabolism disorders and intestinal microflora disorders also play an important role in the occurrence and development of CI.

## 8 INTERVENTION OF CI BY REGULATION OF BLOOD LIPIDS

CI is a complex pathophysiological process for which there are no effective prophylactic and therapeutic measures. Some active interventions to reduce the risk factors of cardiovascular and cerebrovascular diseases can reduce the incidence of CI and delay its onset, such as stabilizing blood pressure and blood sugar, quitting smoking and alcohol, adjusting the mood and psychology.^66^ Abnormal blood lipid levels are an important risk factor for cardiovascular and cerebrovascular diseases, and as a result, controlling blood lipid levels is important in the prophylaxis of CI. Generally, hyperlipidemia can be regulated by diet therapy, such as by avoiding greasy and high‐cholesterol foods, and intake of more vegetables and fruits. If diet therapy fails, lipid‐lowering drugs can be prescribed.^67^

At present, there are multiple lipid‐lowering drugs with various pharacological mechanisms. Common lipid‐lowering drugs include statins, fibrates, niacin, omega‐3 fatty acids, linoleic acid, and bile acid integrators. Among them, statins are the most widely used, in spite of the fact that controversies remain over their effects on CI. Statins are selective 3‐hydroxy‐3‐methylglutaryl coenzyme A (HMG‐CoA) reductase inhibitors, which can enhance HDL‐C levels by promoting cholesterol synthesis and reducing the formation and release of LDL‐C by inhibiting its activity.^68^ As for patients with hyperlipidemia, statins can not only regulate lipid metabolism but also enable prevention and management of hyperlipidemia‐related complications. It is reported that statin treatment can induce better improvements in cognitive function in CI patients with lipid metabolism disorders than those who do not consume statins, especially atorvastatin and lovastatin.^69^ As per a systematic review analysis, statins can reduce the risk of AD and MCI, but with no effect on VaD.^70^ For patients whose LDL‐C levels are already very low, statins can further reduce LDL‐C levels and cardiovascular risks.^71^ In addition, statins can improve the function of endothelial cells by reducing the production of reactive oxygen species and enhancing that of NO.^72^ They can not only inhibit key enzymatic reactions that lead to amyloid deposition and plaque formation by reducing serum cholesterol levels^73^ but also protect brain functions by promoting the transformation of microglia activation into the anti‐inflammatory phenotype and alleviating brain atrophy.^74^ Therefore, statins are currently considered as safe, well‐tolerated, and effective drugs for the treatment of hypercholesterolemia.

Nevertheless, there are also some negative impacts of statins on CI. According to a retrospective analysis,^75^ the long‐term use of statins can increase the risk of CI because it leads to excessive inhibition of TG, damage of myelin formation, induction of cerebrovascular diseases, which leads to impaired cognitive function. Besides, it has been reported in another study that the administration of statins induces symptoms of forgetfulness, memory loss, and delusions, which are not severe and can be reversed after drug withdrawal. So far, there is still a lack of evidence to confirm the negative influence of statins on CI.^76^, ^77^, ^78^ It has been suggested in some studies that statins do not influence CI. As revealed by the findings of Eshaghi et al.,^79^ a 2‐year administration of simvastatin significantly improved the frontal lobe function in patients with progressive multiple sclerosis, although it failed to significantly improve cognitive function compared with the placebo group. It is suggested that the administration of simvastatin may not influence the cognitive function in patients with progressive multiple sclerosis. Besides, it has been demonstrated in a study by Trigiani et al.^80^ that although simvastatin treatment can reduce plasma TG and LDL‐C levels, it does not significantly change the levels of TG and LDL‐C, as well as Aβ deposition in cerebrospinal fluid.

Collectively, the regulation effect of blood lipids by statins on CI remains controversial, and requires further exploration.

## 9 SUMMARY AND PROSPECT

Lipid metabolism disorders are closely related to CI. Many lipid metabolism indexes in the human body play a role in the occurrence and development of CI, which can be used to guide the clinical prophylaxis and management of CI. However, there is currently a lack of a consensus on the involvement of TGs in CI, and research on the underlying mechanisms of lipid metabolism indexes in improving CI is still in the infancy stage. Besides, the effective measures and mechanisms for the regulation of lipid metabolisms in the prophylaxis and management of CI remain unclear. The above‐mentioned issues should be comprehensively explored in the future, through which the occurrence and development of CI can be controlled, and hazards to the physical and mental health of affected people can be reduced.

## CONFLICT OF INTERESTS

The authors declare that there are no conflicts of interest.

## ETHICS STATEMENT

Not applicable.

## AUTHOR CONTRIBUTIONS

Min Zhang conceived and wrote the article under the guidance of Yu‐Hang Zhu, while Zhao‐Qiong Zhu guided and helped with the writing of the article and completed the final draft with the help of all the authors. Zhao‐Qiong Zhu approved the final draft of the paper.

## TRANSPARENCY STATEMENT

All the authors affirm that this manuscript is an honest, accurate, and transparent account of the study being reported; that no important aspects of the study have been omitted; and that any discrepancies from the study as planned (and, if relevant, registered) have been explained.

## DATA AVAILABILITY STATEMENT

Data sharing not applicable to this article as no datasets were generated or analysed during the current study.
